# Thrombin Activatable Fibrinolysis Inhibitor (TAFI): An Updated Narrative Review

**DOI:** 10.3390/ijms22073670

**Published:** 2021-04-01

**Authors:** Machteld Sillen, Paul J. Declerck

**Affiliations:** Laboratory for Therapeutic and Diagnostic Antibodies, Department of Pharmaceutical and Pharmacological Sciences, KU Leuven, B-3000 Leuven, Belgium; machteld.sillen@kuleuven.be

**Keywords:** thrombin activatable fibrinolysis inhibitor, TAFI, coagulation, fibrinolysis, proCPU, proCPB, proCPR, carboxypeptidase

## Abstract

Thrombin activatable fibrinolysis inhibitor (TAFI), a proenzyme, is converted to a potent attenuator of the fibrinolytic system upon activation by thrombin, plasmin, or the thrombin/thrombomodulin complex. Since TAFI forms a molecular link between coagulation and fibrinolysis and plays a potential role in venous and arterial thrombotic diseases, much interest has been tied to the development of molecules that antagonize its function. This review aims at providing a general overview on the biochemical properties of TAFI, its (patho)physiologic function, and various strategies to stimulate the fibrinolytic system by interfering with (activated) TAFI functionality.

## 1. Introduction

Hemostasis is an essential process to safeguard the patency of the vascular system and the surrounding tissues and requires a delicate balance between the formation (coagulation) and the dissolution (fibrinolysis) of blood clots. Upon vascular injury, the coagulatory response is initiated, ultimately resulting in a thrombin burst, which plays a key role in the formation and stabilization of the fibrin clot as well as in the protection of this clot from degradation through the activation of thrombin activatable fibrinolysis inhibitor (TAFI) [1,2]. Normally, the coagulatory response is balanced by the action of the plasminogen activator/plasmin system [3]. The key fibrinolytic enzyme, plasmin, dissolves the blood clot by degrading the fibrin meshwork into soluble fibrin degradation products and exposing new carboxy-terminal (C-terminal) lysines at the fibrin surface, which serve to mediate a positive feedback mechanism in the fibrinolytic process by (I) promoting the binding of plasminogen and therefore also its activation to plasmin by tissue-type plasminogen activator (tPA) [4] and (II) by binding plasmin and thus protecting it against its major plasma inhibitor α_2_-antiplasmin [5]. To prevent hyperfibrinolysis, the action of plasmin is negatively modulated at different levels. Firstly, at the level of plasminogen activation by plasminogen activator inhibitor-1 (PAI-1), which is the main physiological inhibitor of tPA and urokinase-type plasminogen activator (uPA) (reviewed in [6,7]). Secondly, at the level of plasmin by α_2_-antiplasmin (reviewed in [8]). Thirdly, at the level of the blood clot by activated TAFI (TAFIa), a zinc-dependent metallocarboxypeptidase that removes the C-terminal lysines from the partially degraded fibrin clot and thereby abrogates the fibrin cofactor function in plasminogen activation (reviewed in [9,10]). As TAFI is being activated by thrombin, the key component of the coagulatory system, and attenuates the fibrinolytic response, TAFI forms an important molecular link between coagulation and fibrinolysis. Since a variety of studies have demonstrated a role for TAFI in thrombotic disorders (reviewed in [11,12]), several small molecule-, peptide-, and antibody-based inhibitors have been designed in order to explore the potential benefit of pharmacological inhibition of TAFI. This narrative review aims at providing a general overview on the biochemical properties of TAFI/TAFIa, the (patho)physiologic role of TAFIa, and various strategies to stimulate the fibrinolytic system by interfering with TAFI functionality.

## 2. Discovery and Nomenclature

Thrombin activatable fibrinolysis inhibitor (TAFI) was first discovered more than three decades ago in 1989 as a novel unstable molecular form of arginine carboxypeptidase activity in fresh serum prepared from human blood. Because of its instability, it was first named unstable carboxypeptidase (CPU) [13]. Shortly after, another independent study reported the identification of an arginine-specific carboxypeptidase (CPR) generated in blood during coagulation or inflammation [14]. In 1991, a third study revealed a plasminogen-binding protein being present in plasma with a similar amino acid sequence to pancreatic carboxypeptidase B and was therefore named plasma procarboxypeptidase B (plasma proCPB) [15]. In 1995, the discovery of a 60-kDa carboxypeptidase zymogen was reported, that upon activation by thrombin exerted antifibrinolytic effects [16]. This protein was accordingly named thrombin activatable fibrinolysis inhibitor (TAFI). Subsequent amino-terminal sequencing revealed that CPU, CPR, plasma proCPB, and TAFI were identical [17]. To emphasize its main physiological function during fibrinolysis (antifibrinolytic) and its connection to the coagulation system (activatable by thrombin), the term thrombin activatable fibrinolysis inhibitor (TAFI) is widely accepted and used to refer to the zymogen.

## 3. TAFI Synthesis and Distribution

The human TAFI encoding gene, *CPB2*, was mapped to chromosome 13 (13q14.11) and contains 11 exons [17,18]. Two out of 19 identified single-nucleotide polymorphisms (SNPs), +505 G/A and +1040 C/T located in the coding region, result in amino acid substitutions 147 Ala/Thr and 325 Thr/Ile, respectively [19]. As a consequence, TAFI exists as four isoforms, i.e., TAFI-A147-T325, TAFI-A147-I325, TAFI-T147-T325, and TAFI-T147-I325, of which the 325 Thr/Ile polymorphism has an impact on TAFIa stability [20].

TAFI is predominantly synthesized in the liver as a preproenzyme containing 423 amino acids and, after removal of the 22-residue long signal peptide, is secreted into the blood circulation as a 56-kDa proenzyme [15]. TAFI circulates in plasma at concentrations ranging from 73 to 275 nM (corresponding to 4–15 µg/mL) [21,22], of which the apparently large interindividual variation can mainly be attributed to the differential reactivity of the 325 Thr/Ile isoforms of TAFI in some commercially available enzyme-linked immunosorbent assays (ELISAs) [23]. Using isoform-independent ELISAs, it was observed that less than 25% of the variation in plasma levels is due to TAFI gene polymorphisms (outside the encoding region) that may modulate gene expression or affect mRNA stability [19,24,25].

Another pool of TAFI is synthesized in the precursors of blood platelets, the megakaryocytes, and is released upon activation of platelets by thrombin, adenosine diphosphate, and collagen [26]. Despite the minute amounts of TAFI stored in platelets, representing 0.1% of total blood TAFI, it was suggested that platelet-derived TAFI may play an important antifibrinolytic role through a local boost of TAFIa activity owing to the high concentration of platelets within the blood clot [26]. Indeed, TAFI secreted from platelets may affect the resistance to fibrinolysis that is conferred upon platelet-rich clots; however, it was recently shown that activation of plasma-derived TAFI, but not platelet-derived TAFI, is essential for the attenuation of fibrinolysis [27]. Activated TAFI (TAFIa) exerts its antifibrinolytic properties through a threshold-dependent mechanism, with the threshold-value being proportional to the plasmin concentration in plasma, which in turn depends on the concentrations of tPA and α_2_-antiplasmin [28,29]. Importantly, only small amounts of TAFIa, i.e., 1% of total TAFI protein, are required to attenuate fibrinolysis [28].

## 4. TAFI Activation and Instability

### 4.1. TAFI Is a Metallocarboxypeptidase

**Figure 1:**
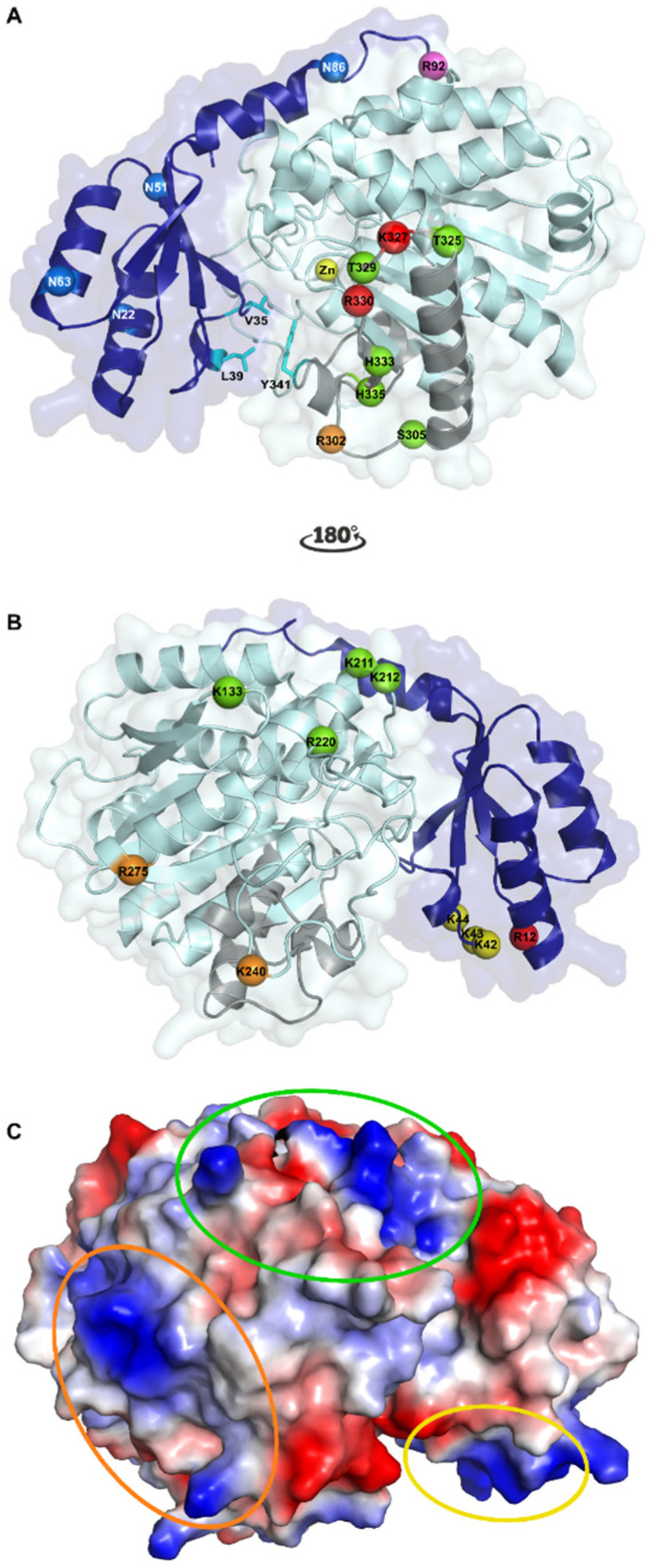
Crystallographic structure of human thrombin activatable fibrinolysis inhibitor (TAFI). (**A**) Cartoon representation of TAFI. The activation peptide (AP) and the catalytic moiety are colored in dark and light blue, respectively. The catalytic zinc-ion in the active center is shown as a yellow sphere. The four glycosylation sites in the AP (Asn22, Asn51, Asn63, and Asn86) are represented by blue spheres. TAFI can be activated through cleavage at Arg92 (shown as a magenta sphere) by thrombin, plasmin, or the thrombin/thrombomodulin complex. Upon the subsequent conformational change to inactivated TAFIa, (TAFIai) a cryptic cleavage site at Arg302 (shown as an orange sphere) becomes exposed and can be cleaved by plasmin or thrombin. Two additional plasmin cleavage sites, Lys327 and Arg330, are indicated by red spheres. Five residues that have been mutated within the dynamic flap region (colored in grey) and result in the most stable TAFIa mutant are indicated by green spheres (Ser305Cys, Thr325Ile, Thr329Ile, His333Tyr, and His335Gln). The dynamic flap, of which the mobility leads to conformational changes that disrupt the catalytic site to form TAFIai, is stabilized by hydrophobic interactions between Val35 and Leu39 of the AP and Tyr341 in the dynamic flap (shown as cyan sticks). (**B**) Binding sites on TAFI for TAFI-activators after rotating panel A by 180° along the y-axis. The three putative thrombomodulin (TM) binding sites, Lys42/Lys43/Lys44, Lys133/Lys211/Lys212/Arg220, and Lys240/Arg275, are indicated by yellow, green, and orange spheres, respectively. Arginine at position 12, which plays an important role in TM-stimulated TAFI activation by thrombin, is indicated by a red sphere and may either constitute a potential cleavage site for thrombin or an exosite for TM. Furthermore, Lys133 may also be a part of the plasmin binding site on TAFI. (**C**) Charged surface representation of TAFI. The three putative TM binding sites are indicated by ovals in the same color as the spheres representing the binding sites in panel B. This figure was generated using the Protein Data Bank structure with PDB ID 3D66 [30]).

Activated TAFI (TAFIa), a member of the metallocarboxypeptidase family, is a zinc-dependent exopeptidase that cleaves carboxy-terminal peptide bonds. The metallocarboxypeptidases are divided into two subfamilies, A and B. TAFI belongs to subfamily A that is characterized by a high structural similarity, i.e., a globular proenzyme consisting of two separate moieties: The activation peptide and the catalytic domain. In this respect, TAFI is a proenzyme that contains a 92-residue long amino-terminal (N-terminal) activation peptide (Phe1-Arg92, 20 kDa, heavily glycosilated) and a catalytic domain of 309 residues (Ala93-Val401, 36 kDa) (Figure 1A).

### 4.2. Three-Dimensional Strucures of TAFI and TAFIa

To date, 17 structures containing human, bovine, or porcine TAFI or TAFIa have been published online in the Protein Data Bank (PDB) (Table 1). The first crystal structure of TAFI was solved in 2008 using TAFI that was recombinantly expressed in a HEK293ES cell line and thus contained a homogenous N-linked glycan profile of the (Man)_5_(GlcNAc)_2_ type [30]. Five putative N-linked glycosylation sites were identified, of which four sites reside within the activation peptide (Asn22, Asn51, Asn63, and Asn86, Figure 1) and one within the TAFIa moiety (Asn219) [31]. However, only the activation peptide was shown to be heavily glycosylated, whereas glycosylation at the Asn219 site seems irreconcilable with the TAFI crystal structure as this residue is completely buried within the catalytic domain [30]. The first 76 residues of the activation peptide (Phe1-Val76) fold into four β-strands and two α-helices that form an open sandwich antiparallel α/β-fold, which is connected by a partially α-helical linker region (Glu77-Arg92) to the catalytic moiety. Both the structures of intact TAFI (PDB ID 3D66 [30]) and TAFIa (PDB ID 3LMS [32]) reveal that the catalytic moiety has a globular shape characterized by a typical α/β-hydrolase fold, comprising an eight-stranded mixed β-sheet flanked by nine α-helices.

### 4.3. TAFI Activation

Even though TAFI exerts low intrinsic carboxypeptidase activity, also referred to as zymogen activity [39], the antifibrinolytic property of TAFI relies on the carboxypeptidase activity of TAFIa [40]. TAFIa is generated upon proteolytic cleavage of the Arg92-Ala93 bond by trypsin-like proteases, such as thrombin or plasmin, resulting in the release of the activation peptide from the catalytic TAFIa moiety [15].

Thrombin is a weak activator of TAFI; however, by forming a complex with either soluble or membrane-bound thrombomodulin (TM), the catalytic efficiency of thrombin-mediated TAFI activation is increased 1250-fold [41,42]. Furthermore, the thrombin/thrombomodulin (T/TM) complex also efficiently generates activated protein C. Activated protein C is both a direct anticoagulant, i.e., by inactivating activated clotting factors V and VIII, as well as indirectly profibrinolytic, i.e., by attenuating prothrombin activation and thus the subsequent TAFI activation. Thus, whereas massive coagulation is prevented through the activation of protein C by T/TM, generation of TAFIa by T/TM results in a protection of the formed clot [43].

Alternatively, TAFI can be activated by plasmin, which is a stronger activator than thrombin [44]. Even though the efficiency of plasmin-mediated TAFI activation is enhanced by glycosaminoglycans such as heparin, the catalytic efficiency of plasmin/heparin remains 10-fold lower than that of the T/TM complex [44]. Therefore, the T/TM complex was postulated to be the main physiological activator of TAFI, as was also suggested by an in vivo study using a monoclonal antibody (mAb) that selectively inhibits T/TM-mediated TAFI activation [45]. In in vitro settings, a biphasic pattern in time associated with thrombin-induced (during thrombin formation) versus plasmin-induced TAFI activation (during the fibrinolytic phase) has been demonstrated [46]. In a consecutive study, the second TAFIa activity peak generated through plasmin-mediated activation could not be translated directly to the fibrinolytic rate [47]. However, other studies using mAbs that mainly impair plasmin-mediated TAFI activation revealed that plasmin contributes to TAFI activation both during clot formation and lysis in vitro [48], and showed to be a relevant physiological activator of TAFI in vivo as well [49]. This therefore indicates that plasmin-mediated TAFI activation may be of direct importance in vivo, where a more dynamic interplay exists between coagulation and fibrinolysis.

Using the crystal structure of thrombin in a complex with thrombomodulin fragments (epidermal growth factor (EGF)-like domains 4, 5, and 6 designated as TM-EGF456) (PDB ID 1DX5 [50]) and a homology model of TAFI that was built based on a crystal structure of human procarboxypeptidase B (PDB ID 1KWM [51]), a structural model of the ternary TAFI/thrombin/TM-EGF456 complex was built [52]. Based on this model and mutagenesis studies, three positively charged surface patches on TAFI, comprising residues Lys42/Lys43/Lys44, Lys133/Lys211/Lys212/Arg220, and Lys240/Arg275, have been suggested as binding sites for the C-loop of the TM-EGF-like domain 3 [52,53]. Furthermore, Arg12, which is located in close proximity to Lys42/Lys43/Lys44, is a potential thrombin cleavage site and plays an important role in TM-stimulated TAFI activation by thrombin [54]. However, at that point, it remained unclear whether cleavage at Arg12 accelerates TM-mediated TAFI activation or whether Arg12 belongs to an exosite for TM. A mutagenesis study later confirmed that both Arg12 and the triple lysine cluster (Lys42/Lys43/Lys44) were critical for the interaction of TAFI with TM as well as for the antifibrinolytic potential of TAFI [55]. Another study employing a deletion mutant of TAFI lacking the first 73 residues of the activation peptide (TAFI-S305C-T325I-T329I-H333Y-H335Q-Δ1–73 or TAFI-CIIYQ-Δ1–73), and thus also lacking Arg12 and the triple lysine cluster, further demonstrated that indeed this N-terminal part of the activation peptide is essential for the co-factor function of TM in accelerating TAFI-activation by thrombin [56]. In contrast, another TAFI mutant TAFI-K133A could be activated by thrombin and the T/TM complex but not by plasmin, indicating that Lys133 may be a part of the plasmin binding site on TAFI [49]. Together, this demonstrates that even though activation of TAFI by thrombin, the T/TM complex, or plasmin involves the same cleavage site at Arg92, these activators bind different residues or regions in the TAFI molecule.

### 4.4. TAFI Instability

Activated TAFI is thermally unstable, and spontaneously converts to an inactive conformation (designated as TAFIai) with a half-life of 8 min (Thr325 polymorphism) or 15 min (Ile325 polymorphism) at 37 °C [57,58]. As no physiological inhibitors of TAFI have been described, this intrinsic instability of TAFIa is thought to play a role in autoregulation of its antifibrinolytic activity in vivo. The crystal structure of intact TAFI (PDB ID 3D66) revealed poor electron density levels and increased B-factors for a segment comprising residues Phe296-Trp350 in the catalytic domain, which is part of the catalytic cleft wall [30]. Because reduced electron density levels are mostly caused by a higher mobility, this region is therefore referred to as the dynamic flap region (Figure 1). In intact TAFI, the activation peptide shields the catalytic pocket, which contains the catalytic zinc ion coordinated by His159, Glu162, and His288, and stabilizes the dynamic flap region through hydrophobic interactions involving residues Val35 and Leu39 of the activation peptide and Tyr341 of the catalytic domain. In a later study, the TAFI deletion mutant TAFI-CIIYQ-Δ1–73 showed similar stability to that of intact TAFI-CIIYQ, whereas cleavage of the Arg92-Ala93 bond leads to the formation of a less stable activated TAFI-CIIYQ. This suggests that, apart from the interactions between the activation peptide and the dynamic flap, the segment Ala74-Arg92 may also contribute to the role of the activation peptide in stabilizing regions in the catalytic domain outside the dynamic flap region in intact TAFI [56]. Activation of TAFI, however, leads to the dissociation of the activation peptide and thus a disruption of these stabilizing interactions, resulting in an increased mobility in the dynamic flap, eventually leading to conformational changes that disrupt the catalytic site.

Interestingly, comparison of the crystal structure of intact TAFI (PDB ID 3D66 [30]) with the crystal structure of TAFI in complex with a carboxypeptidase inhibitor, (2-guanidinoethylmercapto)-succinic acid (GEMSA) (PDB ID 3D67 [30]) revealed that the dynamic flap is stabilized upon binding of GEMSA within the active site. Apart from being more stable at lower temperature [57], several TAFI mutants have been reported that result in a remarkable stabilization of TAFIa [59,60,61]. These mutants contain either four (TAFI-T325I-T329I-H333Y-H335Q or TAFI-IIYQ, PDB ID 3D68 [30]) or five (TAFI-S305C-T325I-T329I-H333Y-H335Q or TAFI-CIIYQ, [61]) stabilizing mutations in the dynamic flap region, stabilizing TAFIa through more extensive interactions between the dynamic flap and the stable core of the catalytic moiety, indicating the important role of this region in TAFIa instability. Importantly, the antifibrinolytic effects of these TAFIa mutants correlate with their increased stability, underscoring the importance of the intrinsic instability in limiting TAFIa activity. Furthermore, upon the conformational change of TAFIa to TAFIai, a cryptic cleavage site at Arg302 becomes exposed [30], resulting in a subsequent irreversible degradation of TAFIai through proteolytic cleavage by thrombin or plasmin [62]. In addition, cleavage of the proenzyme TAFI at Lys327 and Arg330 by plasmin results in an inactive 45 kDa fragment [63].

## 5. (Patho)Physiological Role of TAFI

Cardiovascular disease and thrombotic disorders are often caused by an increased coagulatory or an impaired fibrinolytic response. Due to the antifibrinolytic activity of TAFIa, elevated levels of TAFI/TAFIa are expected to generate a hypofibrinolytic state and constitute a potential risk factor for various thrombotic diseases. Furthermore, studies have shown that the SNPs in the TAFI gene contribute to plasma TAFI concentrations and may thus also contribute to a higher risk for these diseases [64,65,66]. Even though mice engineered to be completely TAFI deficient by gene targeting did not display any observable phenotype [67], another study demonstrated that a significant reduction in thrombus formation was observed in TAFI-deficient mice upon FeCl_3_-induced vena cava thrombosis, indicating that TAFI may still play an important physiological role [68]. In this respect, several studies investigated the role of TAFI levels or the TAFI gene polymorphism as risk factors for the development of cardiovascular disease (extensively reviewed in [12]).

Even though several studies could provide a link between TAFI gene SNPs and cerebral venous thrombosis [69], venous thromboembolic disease [70,71], myocardial infarction [72,73], stroke [74], and coronary heart disease [75,76], a clear link remains controversial as it could not be established by many other studies [77,78,79,80,81,82]. Similarly, independent of the contribution from TAFI gene SNPs, ample evidence has been provided of a link between elevated TAFI levels and venous thromboembolic disease [83,84], deep vein thrombosis [85], stroke [82,86,87], and coronary heart disease [88]. On the other hand, whereas in France carriers of the 505A allele (i.e., the Thr147 isoform), which is associated with higher TAFI levels, showed an increased risk of coronary heart disease; this risk was decreased in carriers of the 505 A allele from Northern Ireland [89]. Similar controversial results were reported in studies in which carriers of SNPs resulting in lower TAFI levels showed an increased risk of deep vein thrombosis [78] and myocardial infarction [90], suggesting a complex relationship between TAFI and thrombotic disease.

Apart from having a profound role as an antifibrinolytic protein, an anti-inflammatory role has been described for TAFIa, as it is able to directly inactivate several inflammatory proteins, such as bradykinin, anaphylatoxins C3a and C5a, thrombin-cleaved osteopontin, and plasmin-cleaved chemerin (reviewed in [91]). Because bradykinin has vasodilating properties, TAFIa may also have a function in blood pressure regulation; however, the physiological relevance of this link is not completely understood as several studies reported conflicting data [92,93,94]. Moreover, TAFIa attenuates the formation of plasmin and it has also been reported that C-terminal lysines and arginines from cellular plasminogen receptors are also substrates of TAFIa [95], therefore suggesting a role for TAFIa in cellular processes involving wound healing, cell migration, and angiogenesis, which also contributes to its anti-inflammatory activity [96,97,98]. However, the exact role of TAFI in inflammation seems to be very complex, since TAFI deficiency in mice has shown to either worsen, have no effect on, or to improve the outcomes in diverse models of inflammatory disease [99]. Moreover, inflammation has been shown to affect hemostasis and may therefore contribute to the atherosclerotic and thrombotic components of cardiovascular disease [100]. Indeed, the high-grade systemic inflammation, which is observed in patients with inflammatory diseases, such as rheumatoid arthritis and inflammatory bowel disease, puts them at greater risk for developing cardiovascular disease [101,102]. Because of the potentially protective role of TAFI in inflammation-related disorders, as demonstrated in mice models of alveolitis, arthritis, and hepatic inflammation, caution must be taken when inhibiting TAFI [103,104,105].

## 6. Inhibition of TAFI Functionality

To date, no physiological TAFIa inhibitors have been found in plasma. However, several small molecules, peptides, and antibody-based inhibitors have been designed and characterized. Owing to its anti-fibrinolytic effect and association with thrombotic tendencies and risk for cardiovascular disease, TAFIa remains a putative drug target. Prevention of TAFI activation and direct inhibition of TAFIa are two potential pharmacological strategies in the development of profibrinolytic drugs.

### 6.1. Synthetic Peptides

Even though no physiological inhibitors of TAFIa have been identified, protein inhibitors that naturally occur in potatoes (potato tuber carboxypeptidase inhibitor, PTCI) [106], leeches (leech carboxypeptidase inhibitor, LCI) [107], and ticks (tick carboxypeptidase inhibitor, TCI) [108] have been described. They are competitive inhibitors of TAFIa, and the crystal structures of TAFIa in complex with TCI revealed their inhibitory mechanism, i.e., binding across the flexible surface segments that form the rim of the active site cleft and penetrating the active site, thereby blocking access of substrates to the active site [32]. Remarkably, a biphasic effect, i.e., prolonging clot lysis at low concentrations and enhancing clot lysis at high concentrations, has been observed for PTCI [106,109]. This phenomenon can be explained by the stabilizing effect of TAFIa inhibitors and the equilibrium between free and inhibitor-bound TAFIa. While free TAFIa is irreversibly inactivated by its thermal instability, inhibitor-bound TAFIa is stabilized by preventing conformational changes that cause inactivation of TAFIa. However, when the TAFIa-inhibitor complex slowly dissociates, TAFIa is released to replenish the free pool. As long as free TAFIa concentrations stay above the tPA-dependent threshold value, fibrinolysis will be attenuated and remains in its initial phase [106].

### 6.2. Small Molecule Inhibitors

Since TAFIa is a zinc-dependent metallocarboxypeptidase, its catalytic activity can be inhibited by chelating agents such as o-phenanthroline and ethylenediaminetetraacetic acid (EDTA) that chelate the essential zinc-ion in the active site [13,15,110]. On the other hand, reducing agents, such as 2-mercaptoethanol and dithiothreitol, can inhibit TAFIa by disrupting the disulfide bonds in the active site of TAFIa (Cys156-Cys169, Cys228-Cys252, and Cys243-Cys257) [13,15,110]. TAFIa is also sensitive to inhibition by small synthetic substrate analogs including organic arginine analogs such as 2-mercaptomethyl-3-guanidinoethylthiopropanoic acid (MERGETPA) and GEMSA or organic lysine analogs such as ε-aminocaproic acid (ε-ACA) [57,110,111]. Even though these inhibitors are most widely used both in in vitro and in vivo studies, the major drawback of these inhibitors is that they also show inhibitory capacity towards other plasma-circulating carboxypeptidases such as carboxypeptidase N (CPN). Apart from their lack of specificity, they are extremely polar, which may limit their oral availability when using them in an in vivo setting. As a consequence, from a drug discovery point of view, many efforts have been devoted to obtain more selective inhibitors of TAFIa with a favorable pharmacokinetic profile.

Several low molecular weight (LMW) inhibitors of TAFIa have been patented (extensively reviewed elsewhere [12]). Most of these inhibitors display a consensus structure consisting of three characteristic groups, i.e., (I) a basic group that mimics the lysine side chain to bind Asp256 at the bottom of the S1′ specificity pocket of TAFIa, (II) a carboxylic acid that corresponds to the C-terminal carboxylic acid of the lysine that it is replacing, and (III) a functional group to coordinate the catalytic zinc ion [112]. These synthetic inhibitors can best be categorized based on the zinc-coordinating functional group, which often contains an imidazole, thiol, phosphonic or phosphinic acid, sulfonamide, or selenium group. Even though several of these LMW inhibitors have entered phase I and phase II clinical studies and showed an excellent safety profile and selectivity towards TAFI, further development was often discontinued due to various reasons, e.g., unfavorable pharmacokinetic properties (low oral bioavailability, short elimination half-life), no superiority over the standard treatment, or for unknown reasons.

### 6.3. Antibodies and Antibody Fragments

Since small synthetic inhibitors often deal with specificity issues, antibodies have become a key tool in drug discovery owing to their specific binding characteristics and amenability to protein engineering. Several monoclonal antibodies (mAbs) have been raised against TAFI in mice and antibody fragments thereof, such as single-chain variable fragments (scFv), have been generated to circumvent immunogenicity problems that are frequently encountered with the murine parental mAb. However, these smaller derivatives often encounter other difficulties such as a reduced binding affinity or a decreased stability. In contrast, variable antigen-binding domains of camelid antibodies, called nanobodies, share a high degree of sequence identity with human variable domains, indicating lower immunogenicity in human, and show excellent binding affinities, a remarkable stability, and solubility in various conditions. The panels of mAbs and nanobodies that were generated to target TAFI can interfere with TAFI or TAFIa activity, TAFI activation, or use a combination of both inhibitory mechanisms (Table 2).

**Figure 2:**
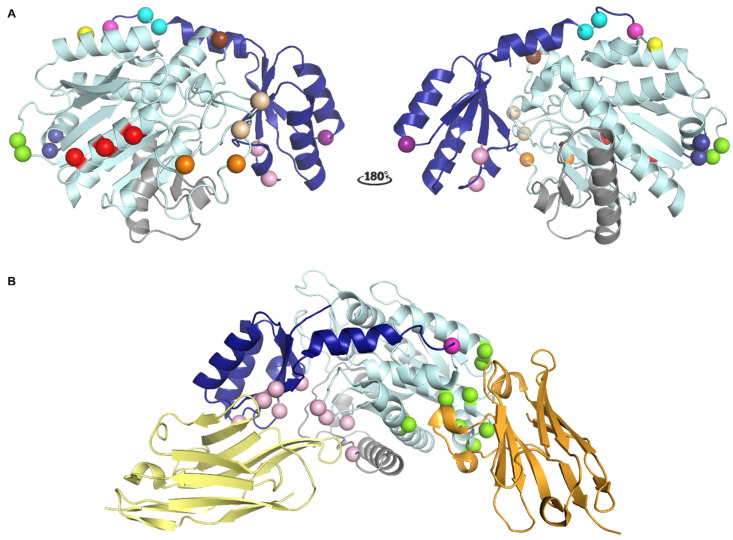
Localization of different epitopes in the structure of TAFI. (**A**) Localization of the epitopes of monoclonal antibodies (mAbs) that interfere with TAFI or TAFIa as determined by mutagenesis studies. The activation peptide (AP) and the catalytic moiety are colored in dark and light blue, respectively. The dynamic flap region is colored in grey. Major determinants of the epitopes are indicated as spheres. The cleavage site at Arg92 is indicated as a magenta sphere. Epitope residues for MA-RT36A3F5 and MA-RT13B2 are indicated by dark blue and light orange spheres, respectively. Epitope residues for MA-RT30D8 and MA-RT82F12 are indicated by the light and dark orange spheres. Epitope residues for nanobody VHH-mTAFI-i49, which destabilizes the TAFI proenzyme, are indicated by the brown and light orange sphere indicated by an asterisk. The epitope residue identified for MA-TCK27A4, which interferes with all modes of TAFI activation, is indicated by a yellow sphere. The epitope residue for MA-T12D11, which selectively inhibits T/TM-mediated TAFI activation, is indicated by a purple sphere. Epitope residues for MA-T94H3 and MA-T1C10, which interfere with both T/TM- and plasmin-mediated TAFI activation, are indicated by pink spheres. Epitope residues for MA-TCK22G2, which interferes with plasmin- and thrombin-mediated TAFI activation, are represented by green spheres. Epitope residues for MA-TCK11A9 and MA-TCK26D6, which mainly inhibit plasmin-mediated TAFI activation, are indicated by red and cyan spheres, respectively. Panel A was generated using the structure of intact human TAFI (PDB ID 3D66). (**B**) Cartoon representation of the crystal structure of TAFI in complex with nanobodies VHH-i83 (yellow) and VHH-a204 (orange) (PDB ID 5HVH). Residues that are engaged in polar interactions with VHH-i83 and VHH-a204 are indicated by pink and green spheres, respectively. The cleavage site at Arg12 is indicated as a magenta sphere.

Within the panel of monoclonal antibodies generated towards rat TAFIa, two mAbs, MA-RT36A3F5 and MA-RT13B2, were found to have a destabilizing effect on TAFIa, thereby shortening its functional half-life [113]. Mutagenesis studies suggested an important role for residues Arg188 and His192 on α-helix 6 in the epitope for MA-RT36A3F5 (Figure 2A). Since α-helix 6 is connected to the active site through α-helix 5, it was hypothesized that binding of MA-RT36A3F5 induces a conformational change, leading to a disruption of the zinc-binding motive in the active site, thereby destabilizing TAFIa. On the other hand, MA-RT13B2 binds on the opposite side of the TAFI molecule with respect to MA-RT36A3F5 and makes interactions with Arg227 and Ser251 located on the loops connecting α-helix 7 with α-helix 8 via the active site residue Arg217 and substrate binding sites Asn234 and Arg235. Apart from destabilizing TAFIa, MA-RT13B2 was also shown to directly interfere with TAFIa activity by reducing the hydrolysis rate of a chromogenic TAFIa substrate. Indeed, by binding to this region in TAFI, MA-RT13B2 may induce a conformational change in the aforementioned loop, which could impact both the stability of TAFIa as well as the accessibility of the active site. The latter is in agreement with the observation that the binding site of MA-RT13B2 was shown to partially overlap with those of two other mAbs within the panel, MA-RT30D8 and MA-RT82F12, which directly inhibit TAFIa activity [113]. Residues Arg227, Ser249, Ser251, and Tyr260 were involved in binding of both mAbs.

Apart from modulating TAFIa activity and destabilizing TAFIa, another concept of TAFI inhibition was discovered with a nanobody, VHH-mTAFI-i49, which transiently stimulates the intrinsic activity of TAFI and simultaneously destabilizes the proenzyme, depleting the pool of activatable TAFI [116]. Epitope mapping revealed that Arg227 and Lys212 belong to the epitope and suggested that binding of the nanobody destabilizes TAFI by disrupting the stabilizing interactions between the activation peptide and the catalytic moiety of PAI-1. Importantly, this hypothesis is in line with the observation that the activation peptide stabilizes regions both within and outside of the dynamic flap of the catalytic moiety [56].

Antibodies that can inhibit T/TM-mediated TAFI activation, either exclusively or in combination with the inhibition of plasmin-mediated TAFI activation, have been shown to bind different regions that do not comprise the cleavage site (Figure 2A) [114]. Binding studies using a human/murine TAFI chimer revealed that the binding sites for these activation-inhibiting mAbs reside in the N-terminal region of the activation peptide of TAFI. Residue Gly66 was identified as a major determinant of the epitope of mAbs, such as MA-T12D11, that exclusively inhibit the T/TM-mediated TAFI activation [114]. Importantly, Gly66 is located on the surface of the protein in close proximity to Arg12 and the Lys42/Lys43/Lys44 region, which was proposed to be important for TM-stimulated TAFI activation by thrombin [52,53]. More recently, structures of TAFI in complex with a nanobody that specifically interferes with TM-dependent TAFI activation, VHH-i83, revealed that this nanobody directly interacts with Arg12, and thereby sterically blocks the binding of TM to the triple lysine cluster within the activation peptide (Figure 2B) [35]. Interestingly, this nanobody also has a direct inhibitory effect on TAFIa; however, only in the presence of the activation peptide [35]. Indeed, the structure of the TAFI-CIIYQ/VHH-i83 complex revealed a previously undescribed mechanism of TAFIa inhibition, i.e., tightly bridging the activation peptide with the catalytic moiety, forming a ternary complex that resembles the inactive proenzyme in which the active site is shielded. On the other hand, important binding residues for mAbs that can interfere with both T/TM- and plasmin-mediated TAFI activation, Val41 (MA-T94H3) and Gln45 (MA-T1C10), are located adjacent to the triple lysine cluster within the activation peptide but at a distance from Gly66 [114].

Antibodies that only inhibit plasmin and/or thrombin-mediated TAFI activation have been shown to bind regions outside the activation peptide (Figure 2A). Monoclonal Ab MA-TCK22G2 was shown to inhibit both plasmin- and thrombin-mediated TAFI activation and presumably binds to residues Thr147 and Ala148 in the loop connecting β-strand 2 and 3 within the catalytic moiety [48]. Monoclonal Ab MA-TCK11A9 was shown to selectively inhibit plasmin-mediated TAFI activation. The major determinants of the MA-TCK11A9 epitope were shown to reside in the α_4_ helix (Lys268, Ser272, and Arg276) in the catalytic moiety of TAFI [48]. Since the epitope residues for MA-TCK22G2 and MA-TCK11A9 are located at a distance from Arg92, it was hypothesized that binding of these mAbs might induce a conformational change or allosteric modulation in the TAFI molecule, preventing plasmin and/or thrombin to activate TAFI. In the case of MA-TCK26D6, which mainly inhibits plasmin-mediated TAFI activation, residues Asp87 and Thr88 located within the activation peptide were shown to contribute to the epitope [49]. It should be noted that this binding site is located close to the Arg92 cleavage site, which is in line with the ability of MA-TCK26D6 to also inhibit thrombin-mediated TAFI activation; however, only to a lesser extent. Apart from the inhibition of plasmin-mediated TAFI activation, a supplemental inhibitory mechanism was revealed, as MA-TCK11A9 and MA-TCK26D6 also have a direct inhibitory effect against TAFIa activity on fibrin [115]. Most interestingly, in the presence of MA-TCK11A9 and MA-TCK26D6, TAFIa was still able to exert its anti-inflammatory role, through inactivation of pro-inflammatory mediators such as thrombin-cleaved osteopontin and C5a. This concept of TAFIa inhibition, leading to a profibrinolytic effect without compromising the strongly intertwined anti-inflammatory role, might therefore be of interest for the development of TAFI inhibitors to treat thrombotic diseases. Notably, due to the in vitro and in vivo potency and cross-reactivity toward rodent TAFI, the scFv fragment of MA-TCK26D6 (scFv-TCK26D6) was developed into a bispecific diabody format together with PAI-1-inhibiting scFv-33H1F7 [118]. Further in vivo evaluation and comparison with the standard thrombolytic therapy showed that the diabody, Db-TCK26D6x33H1F7, holds great promise in both the prevention and treatment of thrombotic disease [119,120]. Alternatively, taking into consideration the numerous advantages of nanobodies over conventional antibody formats, such as higher stability, lower immunogenicity, and better tissue and clot penetration, it might be of interest to pursue a similar dual-targeting strategy using bispecific nanobody constructs comprising one anti-TAFI and one anti-PAI-1 nanobody.

## Figures and Tables

**Table 1:** List of X-ray crystallographic structures containing TAFI or activated TAFI (TAFIa) in the Protein Data Bank (PDB).

Form	PDB ID	TAFI Variant ^1^	Ligand ^2^	Resolution (A)	Ref.
**TAFI**	3D66	Human TAFI	-	3.1	[30]
	3D67	Human TAFI	GEMSA	3.4	[30]
	3D68	Human TAFI-IIYQ	-	2.8	[30]
	4P10	Human TAFI	Compound **5**	2.0	[33]
	3DGV	Bovine TAFI	-	2.5	-
	3OSL	Bovine TAFI	TCI	6.0	[34]
	5HVF	Human TAFI-CIIYQ	Nanobody VHH-i83	2.8	[35]
	5HVG	Human TAFI-CIIYQ	Nanobody VHH-a204	3.0	[35]
	5HVH	Human TAFI-CIIYQ	VHH-i83 + VHH-a204	3.0	[35]
**TAFIa**	3D4U	Bovine TAFIa	TCI	2.5	[36]
	3LMS	Human TAFIa	TCI	1.7	[32]
	4UIA	Porcine TAFIa	Compound **3a**	2.2	[37]
	4UIB	Porcine TAFIa	Compound **3p**	1.9	[37]
	5LYF	Porcine TAFIa	Urea lead of compounds **1** and **6**–**7a**	2.0	[38]
	5LYD	Porcine TAFIa	Compound **1**	2.0	[38]
	5LYI	Porcine TAFIa	Compound **7a**	1.6	[38]
	5LYL	Porcine TAFIa	Compound **6a**	1.8	[38]

^1^ TAFI-IIYQ: TAFI-T325I-T329I-H333Y-H335Q; TAFI-CIIYQ: TAFI-S305C-T325I-T329I-H333Y-H335Q. ^2^ GEMSA: (2-guanidinoethylmercapto)-succinic acid; TCI: tick carboxypeptidase inhibitor.

**Table 2:** Non-exhaustive list ^1^ of monoclonal antibodies (mAbs) and nanobodies (Nbs) that target TAFI or activated TAFI (TAFIa).

		Mechanism	Epitope Residues	Ref.
**mAbs**	MA-RT36A3F5	Destabilizes TAFIa	Arg188, His192	[113]
	MA-RT13B2	Destabilizes TAFIa;	Arg227, Ser251	[113]
		Directly inhibits TAFIa		
	MA-RT30D8	Directly inhibits TAFIa	Arg227, Ser249, Ser251, Tyr260	[113]
	MA-RT82F12	Directly inhibits TAFIa	Arg227, Ser249, Ser251, Tyr260	[113]
	MA-TCK27A4	Inhibits P-, T-, and T/TM-mediated TAFI activation	Phe113	[48]
	MA-T12D11	Inhibits T/TM-mediated TAFI activation	Gly66	[114]
	MA-T94H3	Inhibits T/TM- and P-mediated TAFI activation	Val41	[114]
	MA-T1C10	Inhibits T/TM- and P-mediated TAFI activation	Gln45	[114]
	MA-TCK22G2	Inhibits P- and T-mediated TAFI activation	Thr147, Ala148	[48]
	MA-TCK11A9	Inhibits P-mediated TAFI activation;	Lys268, Ser272, Arg276	[48,115]
		Directly inhibits TAFIa activity on fibrin		
	MA-TCK26D6	Inhibits P-mediated, and to a lesser extent, T-mediated TAFI activation;	Asp87, Thr88	[49,115]
		Directly inhibits TAFIa activity on fibrin		
**Nbs**	VHH-mTAFI-i49	Stimulates TAFI activity	Arg227, Lys212	[116]
		Destabilizes TAFI		
	VHH-a204	Inhibits P-, T-, and T/TM-mediated TAFI activation	Arg117, His118, His175,	[35,117]
			Gln178, Ile182, Gln184,	
			Tyr186, Arg384	
	VHH-i83	Inhibits P- and T/TM-mediated TAFI activation;	Arg12, Gln33, Gln45, Ser70,	[117]
			Val71, Gln292, Arg330,	
		Directly inhibits TAFIa	Thr367, Thr369	

^1^ This list only includes mAbs and Nbs for which the epitope has been mapped my mutagenesis or X-ray crystallographic studies.

## Data Availability

Not applicable.

## Abbreviations

C-terminal	Carboxy-terminal
CPN	Carboxypeptidase N
CPR	Arginine-specific carboxypeptidase
CPU	Unstable carboxypeptidase
EGF	Epidermal growth factor
ELISA	Enzyme-linked immunosorbent assay
GEMSA	(2-guanidinoethylmercapto)-succinic acid
LCI	Leech carboxypeptidase inhibitor
LMW	Low molecular weight
mAb	Monoclonal antibody
N-terminal	Amino-terminal
PAI-1	Plasminogen activator inhibitor-1
PDB	Protein Data Bank
proCPB	Procarboxypeptidase B
PTCI	Potato tuber carboxypeptidase inhibitor
scFv	Single-chain variable fragment
SNPs	Single-nucleotide polymorphisms
TAFI	Thrombin activatable fibrinolysis inhibitor
TAFIa	Activated thrombin activatable fibrinolysis inhibitor
TAFIai	Inactivated conformation of activated thrombin activatable fibrinolysis inhibitor
TAFI-CIIYQ	TAFI-S305C-T325I-T329I-H333Y-H335Q
TCI	Tick carboxypeptidase inhibitor
TM	thrombomodulin
tPA	Tissue-type plasminogen activator
T/TM	Thrombin/thrombomodulin
uPA	Urokinase-type plasminogen activator
